# The Etiology of Bowel Affections in Young Children

**Published:** 1879-10

**Authors:** N. S. Davis

**Affiliations:** Chicago


### ARTICLE III.

### THE ETIOLOGY OF BOWEL AFFECTIONS IN YOUNG CHILDREN.

By N. S. DAVIS, M.D., of Chicago.

In the *Medical and Surgical Reporter* of Philadelphia, for August 2nd, and August 9th, is a very interesting and valuable article by Dr. T. S. Sozinsky of that city, on "the relations of age and the seasonal death rate." The writer makes a careful and reliable analysis of the mortality records of Philadelphia for three successive years including the year 1877; giving the mortality for each month and for each period of life, from birth to one hundred years and over. The tables give the average mortality of each month for the three years, and the same for each month of 1877 separately. The influence of season of the year in relation to age, is most strikingly manifested during the first year of life, but rapidly diminishes with each additional year until from ten to fifteen, the death rate remains the most uniform throughout all the months, but from fifteen onward the variations again increase until the period of old age. For instance, the lowest average mortality in Philadelphia for three years during the first year of life was 203.6 in November, and the highest 877.3 in July; from ten to fifteen the lowest average was 18.6 in October, and the highest, 30.6 in July. We thus see that the mortality during the first year of life is more than four times greater in July than in November, while from ten to fifteen years of age it is less than twice as great in July as in October. If instead of comparing particular months we take groups of months as they constitute spring, summer, autumn and winter, the result will be as follows, taking the average mortality for three years: The average per month for the spring months, March, April and May, under one year of age, is 295.2; for the summer months, June, July and August, 602.3; for the autumn months, September, October and November, 246.8; and for the winter months, December, January and February, 248.2. If we make the same comparison concerning the mortality between the ages of ten and fifteen it will stand as follows: Average for the three spring months, 24.8; for the three summer months, 26.2; for the three autumn months, 20.2; and for the three winter months, 23.1. The same comparison applied to what should be the most stable period of adult life from forty to fifty years of age, gives the following result: Average mortality for the three spring months, 127.4; for the three summer months, 107.9; for the three autumn months, 109.8; and for the three winter months, 111.4. Once more, if we extend the comparison to the period fairly representing old age, that is from seventy to eighty years, the result will be as follows: Average mortality for the spring months, 103.9; for the summer months, 82.1; for the autumn months, 77.6; and for the winter months, 95.8. It is thus seen that the influence of season of the year over the continuance of life is much more strikingly exhibited during infancy and early childhood, than during any part of adult life, or even old age. It is further seen that the mortality during the first period, begins to increase with the commencement of the warm season, reaches its maximum during the highest heat of summer, and recedes to its minimum at the beginning of the cold season, while during both adult life and old age, the mortality begins to increase with the ushering in of winter, reaches its maximum in the spring, and recedes to its minimum during the summer and autumn.

Dr. Edward Smith, in his work on Health and Disease, after a careful study of the vital statistics of England in comparison with meteorological conditons, arrives at the conclusion that the cause of seasonal variations in the prevalence of disease and death, "is the varying degree of vital action proceeding within the body at different seasons of the year." He claims that the maximum of vital activity is in the spring, its minimum in the summer, and its medium or most uniform period in the autumn and winter. While this view might be countenanced by the foregoing statistics in regard to the mortality of young children, the maximum of which corresponds with Dr. Smith's season of supposed minimum of vital activity, it would be directly contradicted by the statistics of mortality from the beginning of adult life to old age, during which, in this country, the statistics show the highest ratio of mortality in the spring. But while Dr. Edward Smith has claimed the spring as the season of greatest vital activity in the human system, Dr. S. Weir Mitchell claims that, in this country, the spring is "the season of greatest depression of health tone." I apprehend that all such general statements as those made by the two eminent investigators just named, are purely theoretical, and of no practical value; and that a more detailed study of the whole subject, more especially in relation to the nature of diseases causing death at different seasons of the year, would show that the variations of mortality in different seasons depended directly on the greater prevalence of diseases dangerous to life at one season than at another. And further, that the causes giving rise to such diseases are peculiar to the seasons, and not to any seasonal differences of internal vital activity or special health tone. Also that the differences in the seasonal mortality at different periods of life, is fully explained by the changes in the relative susceptibility of the several organs and tissues as we advance from infancy to old age. Thus, the fact is familiar to every intelligent reader, that the great increase of mortality which occurs during the two warmest months of every summer in infancy and early childhood, is caused entirely by diseases affecting the alimentary canal, more especially diarrhœa and cholera infantum; while throughout the whole of adult life, the maximum of mortality reached in the winter and spring, is caused almost as exclusively by diseases affecting the respiratory organs, such as pneumonia, pleurisy, bronchitis, laryngitis and phthisis pulmonalis.

Vital statistics and common observation alike show, that while in infancy and early childhood, the digestive organs and the cerebro-spinal nervous structures, are extremely susceptible to the influences of morbific causes, and constitute the seat of a large majority of the diseases fatal to life, such susceptibility rapidly diminishes with the advance in age, while relatively that of the respiratory, circulatory and excretory organs increases until during both adult life and old age, these latter become the seat of a large proportion of the diseases destructive to life. If we keep these changes in the relative susceptibilty of the various organs and structures at different periods of life in view, and the special meteorological characteristics of each season of the year, we shall have no difficulty in understanding the reasons for the seasonal variations in mortality, and the differences in such variations at different periods of life. But we shall never arrive at an exact knowledge of the relations between meteorological conditions and the prevalence of diseases, by a study of *mortality* records alone in comparison with coincident meteorological tables, simply because a large proportion of deaths occurs at periods more or less remote from the date of beginning of the disease causing the deaths. For example, many cases of infantile diarrhœa beginning under certain meteorological conditions early in July, do not terminate fatally until in the latter part of August or even in September, yet the meteorological conditions present and influencing the attack, have undergone many changes before the fatal result is reached.

So also, a pneumonia, pleurisy or bronchitis commencing under certain meteorological conditions in January, may not terminate fatally until in February or March, when the meteorological conditions are entirely different. It follows, therefore, that it is not the records or statistics of mortality, but carefully kept records of the *beginnings* of all acute diseases which are needed to compare with the meteorological records for the purpose of ascertaining how far any given meteorological conditions can be properly considered as efficient causes of disease. To show what has been done concerning this kind of records in relation to the bowel affections of children, I quote the following from a report made by me in the medical section of the American Medical Association at its last annual meeting.*

Referring to the group of diseases known as cholera morbus, cholera infantum, and serous diarrhœa, the report says:

"The general facts in relation to the prevalence and fatality of this group of diseases, are: First, that they are far more destructive to life in infancy than at any subsequent period of human life. Second, that they prevail almost exclusively during the warmest months of the year. Third, that they are far more

---

* See report on the Prevention of Bowel Affections, both in children and adults, as indicated by a comparison of clinical and meteorological facts relating to their etiology, in the transactions of the American Medical Association for 1879.

prevalent over that geographical belt or climatic zone of the earth's surface characterized by a wide range between the extremes of cold in winter and of heat in summer. In our country, this includes all north of the north line of the Gulf States and east of the Rocky Mountains, and excludes the country bordering on the Pacific Ocean and the Gulf of Mexico. These general facts are proved by statistics of mortality familiar to all. For instance, in regard to the first, we find in the city of Chicago 743 deaths attributed to cholera morbus in 1876, of whom 722 were infants; in 1877, the number was 552, of whom 530 were infants; and the same ratio will be found in nearly all the cities and populous towns throughout the belt or climatic zone indicated.

In reference to the second general fact stated, the same tables of mortality show that almost the entire number of deaths attributed to cholera morbus and cholera infantum took place in the months of July, August, and September. Thus in 1876, the total mortality of children under *two* years of age was in June, 238; July, 669; August, 679; September, 350; and October, 222; the entire excess of the mortality of the months of July, August and September over the months of June and October being from cholera morbus, cholera infantum, and diarrhœa. The same result is shown every year. Thus in 1877 the total mortality of *children* under *two* years of age, was, in June, 208; July, 700; August, 523; September, 309; and in October, 217.

The third general fact has not been so generally recognized. Yet, if we compare the infant mortality from bowel complaints in the cities bordering on the Gulf of Mexico and on the Pacific coast with those of the Middle and Northern parts of the United States east of the Rocky Mountains, the effect of extremes of the seasons will be very apparent. Thus, according to the statistics furnished by Dr. Henry Gibbons, Jr., Health Officer for San Francisco, in 1873, the ratio of deaths from cholera infantum in children under *two* years of age was in San Francisco only 5 in every 10,000 of the population, and in New Orleans 7; while in the same year it was 24 in Boston, 25 in Brooklyn, 34 in Chicago, and 21 in Omaha.

The foregoing general facts in relation to the mortality from choleraic and diarrhœal affections, are sufficient to show a close relationship between their prevalence and the heat of summer; and to render it probable that high temperature is, at least, a predisposing cause. But our clinical and meteorological records have developed two additional facts of great importance.

First, that the initial symptoms marking the commencement of each individual case of cholera morbus, cholera infantum, and serous diarrhœa, do not manifest themselves equally from day to day throughout the months of July and August, as the statistics of deaths would imply. On the contrary, the attacks occur in groups, the number of cases multiplying rapidly from three to six or seven days, and then leaving an interval of several days without the occurrence of a single new case. Thus of every hundred cases that occur between the last week in June, in the northern and eastern part of the United States, and the end of July, ninety will have had their initial beginning in groups embracing not more than one-third of the number of days in that month. And of those met with in August and September, more than three-fourths of the cases will have had their beginning during the same group of days in July. Second, a careful examination of the meteorological records kept by the Signal Service Bureau, at the several stations in the northern, middle and eastern parts of the United States shows that the high temperature of every summer manifests itself in waves lasting from three to twelve or fourteen days, but not often exceeding six or seven days consecutively, during which the temperature continues high both day and night. In the northern climatic belt of this country, the first of these waves of high temperature, continuing day and night for more than three days in succession, pretty uniformly manifests itself between the 20th of June and the 10th of July; and just as uniformly marks the *beginning* of the prevalence of choleraic and diarrhœal affections for that summer. These special waves of heat will generally be repeated twice during the month of July, and, with less intensity, once in August. But they vary much both in intensity and duration in different years. By comparing the clinical records furnished at Omaha, Davenport, Cairo, Chicago and Dunkirk, concerning the date of the initial symptoms of cases of the bowel affections under consideration, with the meteorological records, I have found the groups of days affording the principal number of attacks to be the same as those included in the special waves of high temperature; thus clearly demonstrating that *continuous high temperature* is, at least, one of the essential elements of *causation* in the production of this class of diseases. I say *continuous* high temperature, because a careful study of the facts shows that it is not simply an extreme heat of one day, or high heat of any number of days attended by cool nights, that determines the prevalence of these diseases; but continuous heat for several days and nights, during which the mercury rises above 30° C. during the day, and does not fall below 20° C. during the night.

Details illustrating these conclusions were given in the report to the section on Practical Medicine, etc., for 1877; which with the further details collected and carefully compared since, fully justify the statement that the chief *factor* in the causation of choleraic and diarrhœal affections, is, *continuous high temperature*, acting upon populations subject to a wide range between the extreme of cold in the winter, and that of heat in summer. And further, that the degree of prevalence and fatality of such diseases will be in direct ratio to the intensity and duration of the heat waves occurring between the middle of June and the middle of August."

The foregoing statements are strongly corroborated by the difference in the prevalence of the diseases under consideration in the same localities in different years. Thus in Chicago in 1876, the whole number of deaths attributed to these bowel affections, was 743; while in 1877 it was only 552. In comparing the meteorological conditions of the two years, it is found that the heat waves during the summer of 1877 were decidedly less intense and of less duration than in 1876. I see the same thing illustrated in London during the years 1878 and 1879. Thus, says Dr. L. P. Yandell, in a recent letter from London, published in the Louisville *Medical News* for September 13th, 1879: "Diarrhœa is the chief source of mortality in England, as it is in America, among young children. During July (1879) 230 fatal cases of infantile diarrhœa occurred; during the hot July of 1878 there were 1,976 innocents carried off by diarrhœa. Malaria is far the most abundant cause of bowel trouble, and quinine is the great remedy for these affections." In the same letter Dr. Yandell informs us that the whole summer of 1879 in London, was the coldest known since 1860, the mean temperature in July being only 58° F., while that of 1878 was above the average. The aggregate number of deaths of children under 5 years of age, was 2,628 more in the latter than in the former season. It will be seen by the foregoing considerations that we deem continuous high temperature, acting upon populations subject to wide contrasts between the temperature of winter and summer, as the efficient cause of the bowel affections, especially of young children.

It will be seen by the last part of the paragraph quoted from the letter of Dr. Yandell, that he regards *malaria* as the efficient cause. But if this were true these diseases should be as prevalent in New Orleans as in Chicago; in San Francisco as in Boston or New York; and their highest prevalence should be when the action of *malaria* is known to be most intense, namely, in August and September, instead of July. I close this article by quoting again from my report in the transactions of the American Medical Association, already alluded to.

"The coincident circumstances that give additional efficiency to the action of heat, are stillness or want of currents in the atmosphere and want of ventilation in dwellings; excess of moisture; and whatever impairs the general tone of the tissues or increases the sensitiveness of the mucous membrane of the alimentary canal. It is the co-existence of imperfect tissue developments, great susceptibility, and much confinement within the stagnant atmosphere of imperfectly ventilated dwellings, with the seasons of high temperature, that make the diseases under consideration so much more prevalent and fatal in infancy, than at any other period of life.

If these observations are correct they are of great practical value; first, in pointing definitely to the times when, and in what direction, sanitary measures should be adopted for restricting the number and severity of the attacks; and, second, the nature of the measures to be adopted, both for the prevention and cure of this class of human ills. It is plain, that so far as the time when preventive measures should be in readiness for practical use, the facts show that they should be in efficient operation coincident with the beginning of the first wave of continuous high temperature in each summer, and that they may be limited mostly to the period of such waves. If the children of the well-to-do are to be sent out of cities and populous towns to elevated, dry and healthy country districts — if those of the poor are to be provided for in parks and barracks in similar country locations as recently suggested by Dr. J. M. Toner and others — or put on board sanitary hospital ships to float in the cooler and purer air of large bodies of water, as practised to some extent in New York, Chicago, and perhaps other places; any and all these measures to act efficiently as preventives, must be in readiness and actually in active operation coincident with the first wave of persistent high temperature, and not, as is too often the case, delayed until the middle or latter part of July, when three-fourths of all who will be attacked during the season have already begun to suffer and many have died. So far as these measures can be made available at the right time they are efficient and valuable. But fiom the very nature of human society they can never be made to reach more than a moderate percentage of those who need them most. And as the great leading object is to lessen the effects of continuous heat upon the tissues of the body, a simple process that could be readily executed in every family, however poor, and yet prove of great efficiency, would be to introduce the custom of having all young children freely bathed every warm, oppressive summer evening, and during the special waves of hot weather, after the bath put to bed with a wet towel around the body to protract the cooling process. Such a custom, simple and inexpensive as it may appear, if generally adopted by the poor and rich alike, and especially if connected with increased attention to the capacity and ventilation of sleeping rooms, would every summer prevent a large proportion of the attacks that would otherwise occur; and would consequently greatly lessen the aggregate of infant mortality."

As already stated, the clinical records concerning the date of initial symptoms or beginning of attacks of the bowel affections, compared with the meteorological records in the same localities kept by the Signal Service Bureau, have demonstrated fully the constant coincidence between waves or periods of persistent high atmospheric temperature and the rapid devolopment of those affections. The only question that remains unsettled, is, whether the persistent high temperature, occurring in the regions already indicated, acts as a *direct exciting* cause of these affections, or *indirectly* by changing the electric and ozonic conditions of the atmosphere. As the records of the Signal Service Bureau do not include observations concerning the two last mentioned forces, there are not in existence the recorded facts necessary for determining the question.

In referring to the Annual Report of the Board of Health of Michigan for 1878, we find the results of observations concerning the ratio of ozone in the atmosphere, made in seven places in that State, indicated by tables and diagrams constructed by Dr. H. B. Baker; but as the daily record is not given, and the localities are not the same as those where our clinical records were kept, we cannot determine whether there is any fixed relation between the maximum of heat waves and the minimum of ozone. But in comparing Dr. Baker's diagrams representing respectively the range of temperature, atmospheric moisture, and ozone for the year 1877, based on the records of six or seven meteorological stations in the State of Michigan, the interesting fact is at once apparent, that the maximum of both temperature and moisture was reached on the 1st of July, and was maintained with but little diminution through that month and August, while exactly the reverse was the case in reference to ozone; the diagram indicating the minimum of the latter during the months of July and August.

CHICAGO, Sept. 22d, 1879.

